# Endangered *Salvia daiguii* Has Evolved Drought Tolerance Through Photosynthetic, Antioxidant, and Respiratory Adaptations: Implications for Ex Situ Propagule Reintroduction

**DOI:** 10.3390/plants15172693

**Published:** 2026-09-02

**Authors:** Can Xie, Luxuan Huang, Shen Rao, Yanbo Huang, Yukun Wei, Hua Xu

**Affiliations:** 1 School of Modern Industry for Selenium Science and Engineering, Wuhan Polytechnic University, Wuhan 430048, China; can.xie@outlook.com (C.X.); 13477820923@163.com (L.H.); raoshen1989@163.com (S.R.); 2 Shanghai Botanical Garden, Shanghai 200232, China; 3 Shanghai Chenshan Botanical Garden, Shanghai 201602, China; yanbocf@163.com

**Keywords:** endangered *Salvia daiguii*, drought tolerance, respiratory enzymes, antioxidation, photosynthetic pigments, osmotic regulation

## Abstract

*Salvia daiguii* is an endangered plant species with medicinal potential, whose ex situ conservation and reintroduction are essential for population restoration. However, drought tolerance, critical to the survival and establishment of reintroduced populations, remains poorly understood for *S. daiguii*. Using PEG-simulated drought treatments (0–15%), we evaluated physiological drought tolerance mechanisms of *S. daiguii* with its drought-tolerant congener *Salvia meiliensis* as a reference. Drought injury indices increased with rising PEG concentrations in both species, yet *S. daiguii* showed stronger drought tolerance across 5–15% PEG, as evidenced by milder injury, less thiobarbituric acid-reactive substances (TBARS), and higher relative water content in aboveground tissues. Though both species exhibited adaptive drought responses, their response patterns and magnitudes differed. At 10% PEG, *S. daiguii* had higher peroxidase (POD) and ascorbate peroxidase (APX) activities. It accumulated more ascorbate (AsA), soluble sugar, and soluble protein at 10% and 15% PEG and maintained advantages in chlorophyll accumulation and respiratory enzyme activation across all PEG treatments. Higher glutathione reductase (GR) activity in *S. daiguii* at 10% and 15% PEG accelerated glutathione (GSH) regeneration, strengthening its antioxidant capacity. Collectively, the superior drought tolerance of *S. daiguii* involves integrated modulation of photosynthetic, antioxidant and respiratory systems, supporting its conservation and reintroduction.

## 1. Introduction

Ex situ conservation in botanical gardens not only protects endangered plants from extinction also provides resources for the restoration of endangered species in the wild [1]. However, this conservation approach carries several risks. For example, limited garden space restricts population expansion of endangered plants, leading to inbreeding, genetic diversity loss, and species degradation, including reduced seed vigor [1,2,3]. Prolonged cultivation under favorable conditions may also cause phenological shifts, such as altered flowering time [2,4], and can erode adaptation to their original habitats, including drought tolerance [1,5]. To mitigate these risks, ex situ endangered plants should be reintroduced to their native or other suitable habitats as early as possible [6]. Before any reintroduction takes place, their tolerance to various abiotic stresses, especially drought, must be comprehensively assessed.

Drought is viewed as one of the key abiotic stress factors. It causes adverse impacts upon plant survival, development, diversity, and productivity [7,8,9,10]. Its occurrence is mainly associated with inadequate precipitation and excessive evaporation [11]. It is reported that approximately 40% of the terrestrial area on Earth is considered arid due to scarce rainfall, high solar radiation, and fluctuating temperature [12]. With global climate change, drought worldwide is predicted to increase in spatial extent, frequency, duration, and intensity [13,14]. At present, many endangered plant species face a higher extinction risk due to drought caused by climatic change worldwide [15,16]. Many failed reintroduction cases of endangered plants were due to neglecting the assessment of their drought tolerance [5]. Thus, drought presents an enormous challenge for successfully reintroducing endangered plant species.

Drought stress typically causes water deficit in plants, thus adversely affecting their multiple physiological processes [17]. Specifically, plants close stomata under drought to avoid excessive water loss; yet, stomatal closure restricts CO_2_ assimilation, inhibiting photosynthesis while accelerating photorespiration [18,19,20]. Moreover, drought-induced stomatal closure impairs leaf transpirational cooling and consequently leads to simultaneous drought and heat stress, making plants more susceptible to damage under progressive global warming [21]. Drought stress is also capable of inhibiting the synthesis of photosynthetic pigments. This triggers a low photosynthetic efficiency [22]. It can also suppress aerobic respiration by slowing down the tricarboxylic acid (TCA) cycle in mitochondria, reducing glucose metabolism and energy supply [23,24,25,26]. Additionally, drought may block the mitochondrial electron transport chain (ETC) [27,28] and suppress photosynthesis [29], resulting in over-accumulation of reactive oxygen species (ROS) in mitochondria [28] and green tissues, respectively [30], which further causes subsequent oxidative damage to plant cells.

Evidence suggests that many plants, including numerous endangered species, have evolved a set of adaptive mechanisms for drought alleviation [31,32,33]. They can reshape root architecture, such as regulating the density and length of taproots, lateral roots, and root hairs, to expand water-uptake surfaces [34,35]. They can also enhance osmotic adjustment by accumulating compatible solutes such as proline, soluble sugar, and soluble protein, thereby promoting water retention and absorption [25]. To sustain energy metabolism under drought conditions, drought-tolerant plants can accelerate aerobic respiration by activating key respiratory enzymes associated with the TCA cycle and the ETC, including fumarase (FM) [36], succinate dehydrogenase (SDH) [37], malate dehydrogenase (MDH) [38], and cytochrome c oxidase (COX) [39]. To protect endomembrane integrity, these plants also activate sophisticated antioxidant systems to scavenge drought-induced ROS. This involves enhanced enzymatic antioxidant activities, including catalase (CAT), peroxidase (POD), superoxide dismutase (SOD), and ascorbate peroxidase (APX) [22], as well as enhanced levels of non-enzymatic antioxidants, such as ascorbic acid (AsA) and glutathione (GSH) [40,41,42]. Concurrently, glutathione reductase (GR) is substantially activated to sustain GSH regeneration under drought stress [40,42].

In 2019, *Salvia daiguii* Y.K. Wei & Y.B. Huang, a perennial herb, was discovered in Zhangjiajie, Hunan Province, China. This species naturally occurs in stone crevices and rocky cliff faces at altitudes of 600–700 m [43,44]. Its wild population comprises no more than 200 individuals, and Wei et al. [43] recommended listing the species as Critically Endangered according to International Union for Conservation of Nature (IUCN) Red List criteria. The species also possesses considerable medicinal potential, owing to a high concentration of antioxidant and antimicrobial constituents such as caffeic acid, rosmarinic acid, and salicylic acid B [45]. Given its medicinal value and endangered status, urgent conservation action is required for this endangered plant.

Ex situ production of *S. daiguii* commenced at Shanghai Chenshan Botanical Garden in 2019. In fewer than seven years, thousands of individuals have been propagated via tissue culture and clonal division, and more than 100 hand-pollinated seeds have been obtained under nursery conditions. Half of these seeds have completed their full life cycle, including germination, flowering, and fruiting. Under controlled favorable conditions, this species and its propagules have been maintained in both greenhouse and outdoor nurseries for six years. Further expansion of the ex situ population is currently constrained by available garden space. To minimize genetic and adaptive risks associated with long-term ex situ cultivation, reintroduction to the wild is under consideration. Consistent with previous successful reintroduction programs [46], pre-assessment of the species’ adaptation to climatic and environmental factors, particularly drought, is indispensable. However, its drought tolerance remains unexplored to date.

Previous work has demonstrated strong heat tolerance in *S. daiguii* [47]. Considering the widespread occurrence of cross-resistance across multiple abiotic stresses in plants, particularly between drought and heat [48,49,50], we hypothesized that this species would exhibit high drought tolerance. To test this hypothesis, we used *Salvia meiliensis* (a close relative with confirmed drought tolerance) as a reference control and assessed the drought tolerance of the two *Salvia* species based on their phenotypic and physiological reactions to PEG 4000-simulated drought stress to assess the feasibility of reintroducing *S. daiguii*.

## 2. Results

### 2.1. Drought Injury Indices of Two Salvia Species Under Different PEG-Induced Drought Stress Conditions

Drought injury indices of *S. daiguii* and *S. meiliensis* increased gradually with increasing PEG concentrations (Table 1). Specifically, the drought injury index of *S. daiguii* was consistently lower than that of *S. meiliensis* at 5%, 10%, and 15% PEG (Table 1). This finding indicated that *S. daiguii* exhibited stronger drought tolerance than *S. meiliensis* under PEG-induced drought stress conditions.

### 2.2. Effects of PEG-Induced Drought Stress on the Relative Water Content in Two Salvia Species

**Figure 1:**
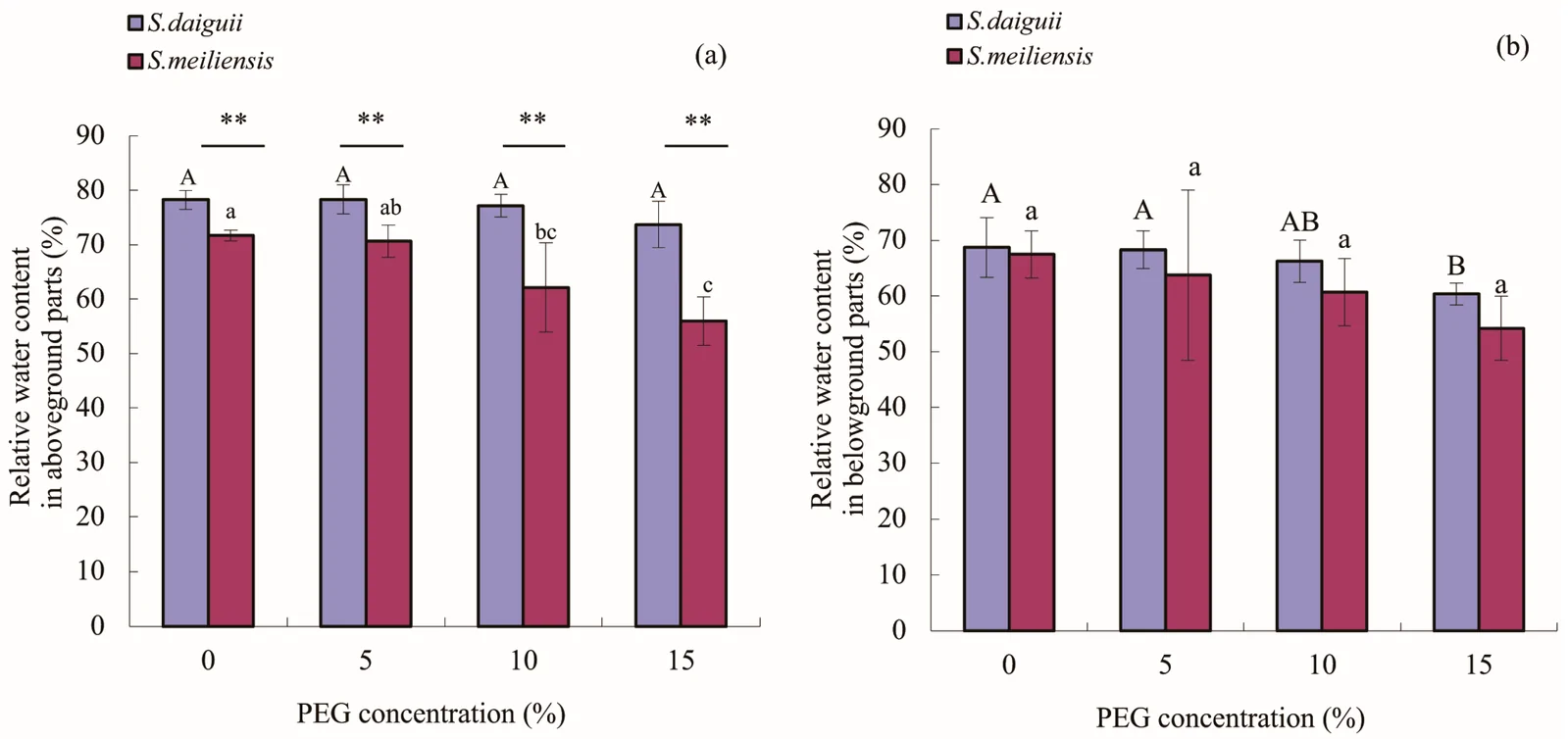
Response patterns of relative water content to PEG-induced drought stress in *S. daiguii* and *S. meiliensis.* (**a**) Relative water content in aboveground tissues; (**b**) Relative water content in belowground tissues. Results are given as mean ± SE (*n* = 5). Distinct uppercase and lowercase letters show significant differences (*p* < 0.05) among various drought treatments for *S. daiguii* and *S. meiliensis*, respectively, as determined by the Tukey test. Double asterisks represent differences at ** *p* < 0.01 between the two species under identical drought conditions, as analyzed by the independent-samples *t*-test.

With increasing PEG concentrations, the relative water content of aboveground tissues in *S. daiguii* did not decrease significantly, whereas this parameter in *S. meiliensis* decreased gradually along the PEG gradient (Figure 1a). For belowground tissues, *S. meiliensis* exhibited no significant reduction in relative water content along the PEG gradient, whereas *S. daiguii* showed a gradual decrease in this parameter (Figure 1b). At 5%, 10%, and 15% PEG, the two *Salvia* species exhibited comparable relative water content in belowground tissues (Figure 1b). In contrast, *S. daiguii* maintained significantly higher relative water content in aboveground tissues than *S. meiliensis* across these three PEG levels (Figure 1a). These results suggested that *S. daiguii* underwent a milder water deficit than *S. meiliensis* under these three PEG-induced drought conditions.

### 2.3. Effects of PEG-Induced Drought Stress on Peroxidation Levels in Two Salvia Species

**Figure 2:**
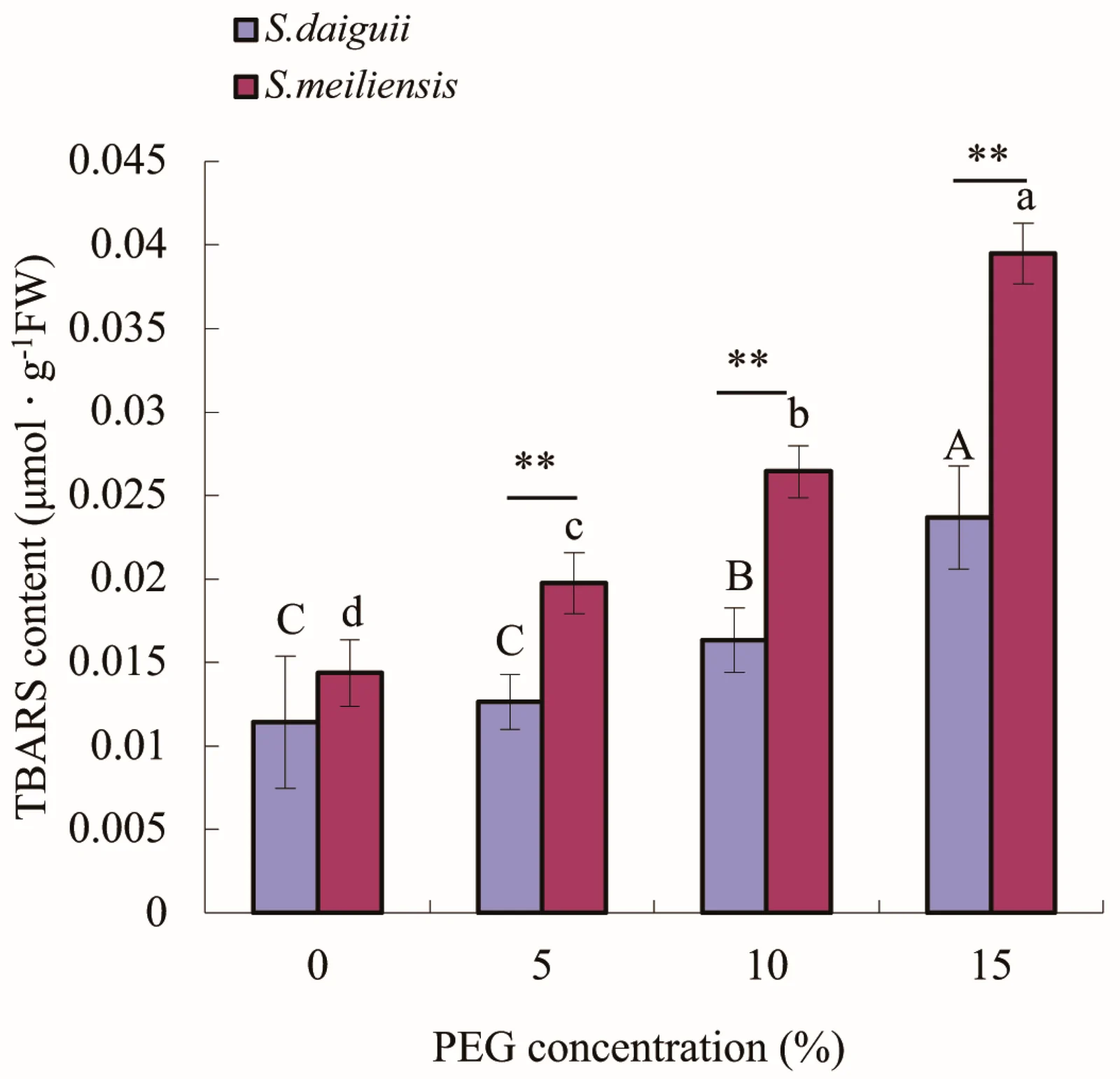
Response patterns of TBARS content to PEG-induced drought stress in *S. daiguii* and *S. meiliensis*. TBARS: thiobarbituric acid-reactive substances. Results are given as mean ± SE (*n* = 5). Distinct uppercase and lowercase letters show significant differences (*p* < 0.05) among various drought treatments for *S. daiguii* and *S. meiliensis*, respectively, as determined by the Tukey test. Double asterisks represent differences at ** *p* < 0.01 between the two species under identical drought conditions, as analyzed by the independent-samples *t*-test. FW: fresh weight.

At 0% PEG, no significant difference in thiobarbituric acid-reactive substances (TBARS) content was observed between *S. daiguii* and *S. meiliensis* (Figure 2), indicating that the two *Salvia* species possessed comparable basal levels of membrane lipid peroxidation under well-watered control conditions. With increasing PEG concentrations, TBARS content in both *Salvia* species showed a gradual upward trend (Figure 2), suggesting that rising levels of PEG-induced drought stress aggravated membrane lipid peroxidation damage. Notably, *S. meiliensis* maintained consistently higher TBARS content than *S. daiguii* at 5%, 10%, and 15% PEG (Figure 2). These results indicated that *S. meiliensis* suffered more severe membrane lipid peroxidation damage than *S. daiguii* under these three PEG-induced drought conditions.

### 2.4. Effects of PEG-Induced Drought Stress upon Chlorophyll Content and Chlorophyll a/b Ratio in Two Salvia Species

**Figure 3:**
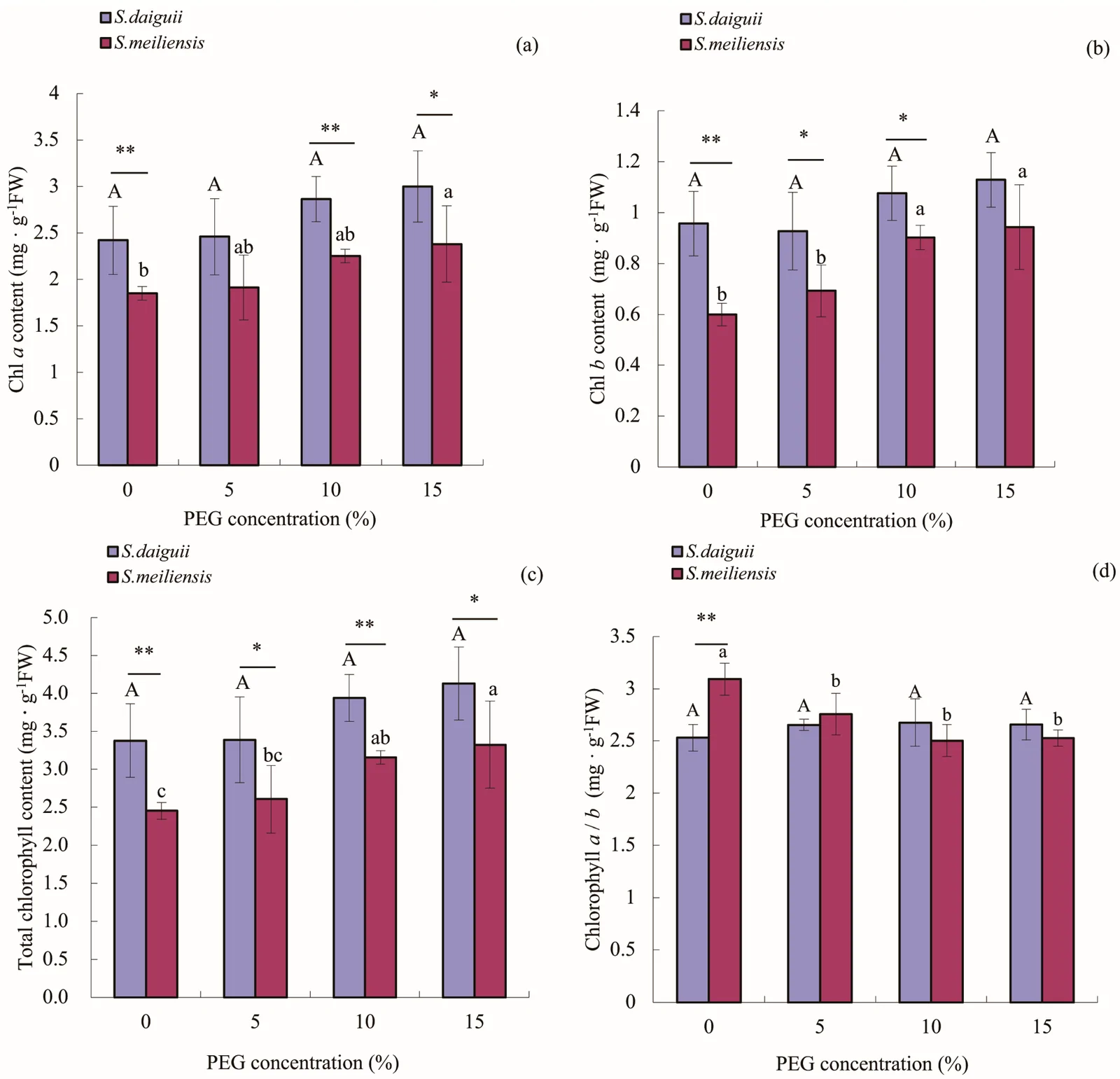
Response patterns of chlorophyll content and Chl *a*/*b* ratio to PEG-induced drought stress in *S. daiguii* and *S. meiliensis*. (**a**) Chl *a* content; (**b**) Chl *b* content; (**c**) Total chlorophyll content; (**d**) Chl *a*/*b* ratio. Chl *a*: chlorophyll *a*; Chl *b*: chlorophyll *b*. Results are given as mean ± SE (*n* = 5). Distinct uppercase and lowercase letters show significant differences (*p* < 0.05) among various drought treatments for *S. daiguii* and *S. meiliensis*, respectively, as determined by the Tukey test. Single and double asterisks represent differences at * *p* < 0.05 and ** *p* < 0.01 between the two species under identical drought conditions, as analyzed by the independent-samples *t*-test. FW: fresh weight.

With increasing PEG concentrations, the content of Chlorophyll *a* (Chl *a*), Chlorophyll *b* (Chl *b*), and total chlorophyll in *S. daiguii* remained relatively stable, whereas these pigment parameters showed a progressive upward trend in *S. meiliensis* (Figure 3a–c). This result indicated that *S. meiliensis* could elevate its photosynthetic pigment level in response to PEG-induced drought stress. Nonetheless, total chlorophyll content in *S. daiguii* was higher than that in *S. meiliensis* at 5%, 10%, and 15% PEG (Figure 3c), suggesting that *S. daiguii* had a greater advantage in chlorophyll synthesis than *S. meiliensis* under these three PEG-induced drought stress conditions.

No significant difference was observed in the Chl *a*/*b* ratio between *S. daiguii* and *S. meiliensis* at 5%, 10%, and 15% PEG. Nevertheless, the two *Salvia* species exhibited divergent response patterns in the Chl *a*/*b* ratio with increasing drought stress levels (Figure 3d). Specifically, the Chl *a*/*b* ratio in *S. daiguii* remained stable with increasing PEG concentrations (Figure 3d), implying that this species could maintain a balanced accumulation ratio between Chl *a* and Chl *b* under the tested drought stress conditions. Conversely, the Chl *a*/*b* ratio in *S. meiliensis* showed a gradual downward trend (Figure 3d), which suggested that this species exhibited greater accumulation of Chl *b* relative to Chl *a* under more severe drought stress.

### 2.5. Effects of PEG-Induced Drought Stress upon Key Enzymes Associated with the TCA Cycle and the Respiratory Chain

**Figure 4:**
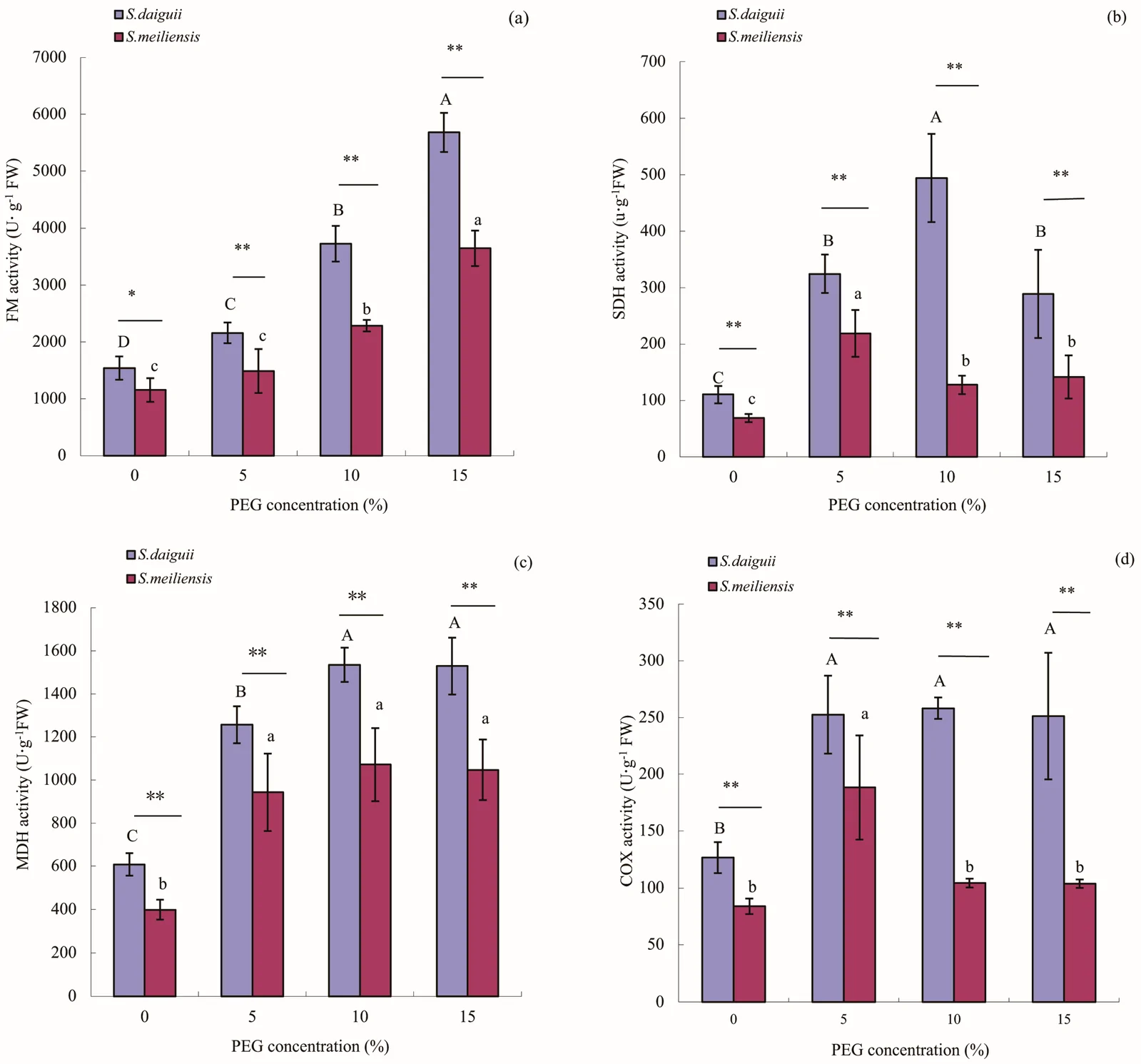
Response patterns of FM, SDH, MDH and COX activities to PEG-induced drought stress in *S. daiguii* and *S. meiliensis*. (**a**) FM activity (FM: fumarase); (**b**) SDH activity (SDH: succinate dehydrogenase); (**c**) MDH activity (MDH: malate dehydrogenase); (**d**) COX activity (COX: cytochrome oxidase). Results are given as mean ± SE (*n* = 5). Distinct uppercase and lowercase letters show significant differences (*p* < 0.05) among various drought treatments for *S. daiguii* and *S. meiliensis*, respectively, as determined by the Tukey test. Single and double asterisks represent differences at * *p* < 0.05 and ** *p* < 0.01 between the two species under identical drought conditions, as analyzed by the independent-samples *t*-test. FW: fresh weight.

With increasing PEG concentrations, FM activity in *S. daiguii* exhibited a steady upward trend (Figure 4a), while its SDH activity showed an initial increase followed by a decline (Figure 4b). Meanwhile, MDH activity in *S. daiguii* increased progressively with elevated PEG concentrations, reached a plateau at 10% PEG, and then maintained this level at 15% PEG (Figure 4c). By contrast, COX activity in *S. daiguii* reached a plateau at 5% PEG and then maintained this level at 10% and 15% PEG (Figure 4d). These results suggested that *S. daiguii* could activate enzymes regarding the TCA cycle and the mitochondrial respiratory ETC under the three tested PEG-induced drought stress conditions.

With increasing PEG concentrations, FM activity in *S. meiliensis* showed a gradual upward trend (Figure 4a), while its MDH activity reached a plateau at 5% PEG and remained unchanged at 10% and 15% PEG (Figure 4c). Meanwhile, SDH and COX activities in *S. meiliensis* showed an initial rise followed by a dramatic decline, with increasing PEG concentrations (Figure 4b,d). Notably, COX activity in *S. meiliensis* at 10% and 15% PEG returned to a level similar to that observed under the 0% PEG control condition (Figure 4d). These results indicated that *S. meiliensis* could activate TCA cycle-associated enzymes under the tested drought stress levels but failed to activate enzymes engaged in the mitochondrial respiratory ETC under 10% and 15% PEG stress.

Overall, the activities of FM, SDH, MDH, and COX in *S. daiguii* were consistently higher than those in *S. meiliensis* under 5%, 10%, and 15% PEG treatments (Figure 4a–d). These findings suggested that *S. daiguii* had a greater advantage than *S. meiliensis* in accelerating the TCA cycle and promoting electron transport within the mitochondrial respiratory chain under these three PEG-induced drought stress conditions.

### 2.6. Effects of PEG-Induced Drought Stress on Non-Enzymatic Antioxidant Content

**Figure 5:**
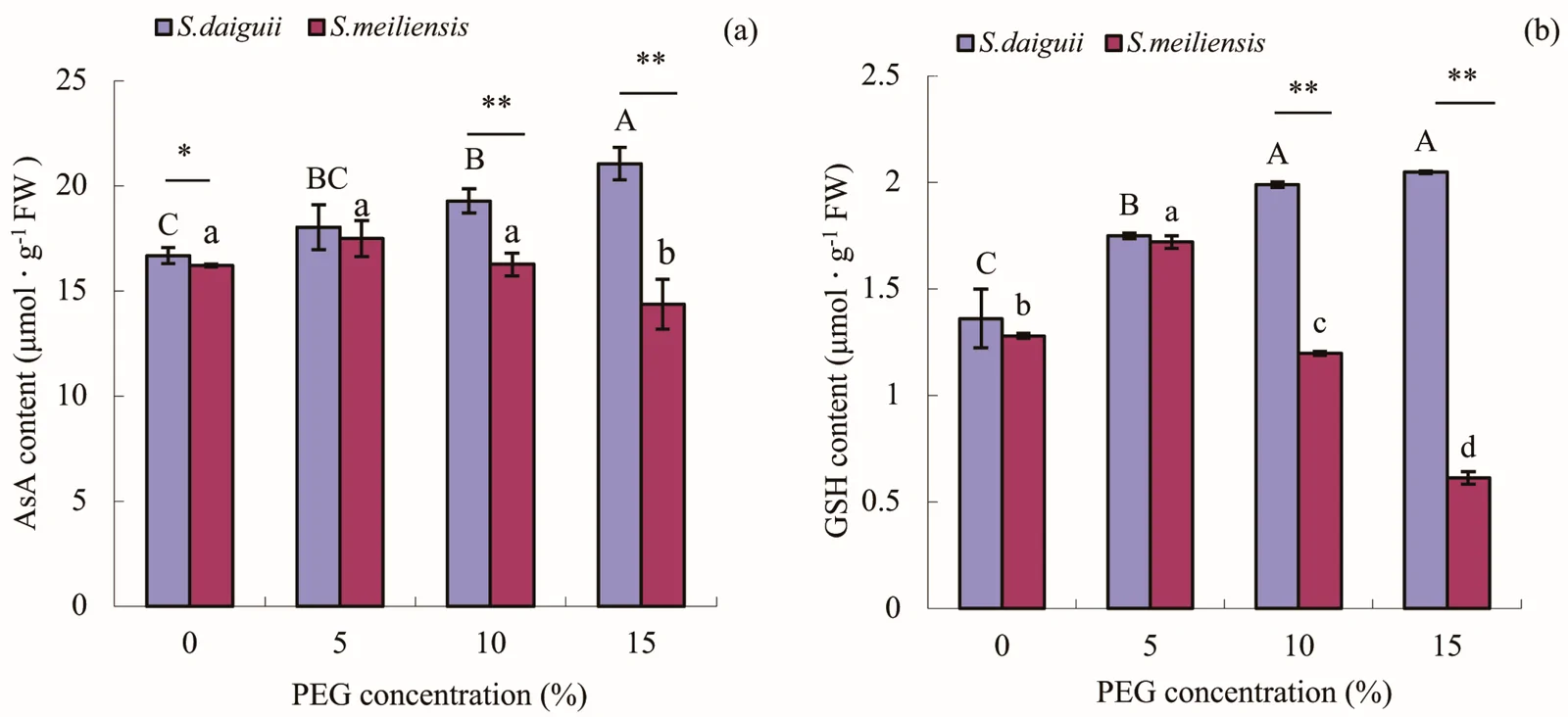
Response patterns of AsA and GSH content to PEG-induced drought stress in *S. daiguii* and *S. meiliensis*. (**a**) AsA content (AsA: ascorbic acid); (**b**) GSH content (GSH: glutathione). Results are given as mean ± SE (*n* = 5). Distinct uppercase and lowercase letters show significant differences (*p* < 0.05) among various drought treatments for *S. daiguii* and *S. meiliensis*, respectively, as determined by the Tukey test. Single and double asterisks represent differences at * *p* < 0.05 and ** *p* < 0.01 between the two species under identical drought conditions, as analyzed by the independent-samples *t*-test. FW: fresh weight.

With increasing PEG concentrations, AsA and GSH content in *S. daiguii* exhibited an upward trend (Figure 5a,b). For *S. meiliensis*, AsA content remained relatively stable from 0% to 10% PEG and decreased markedly only at 15% PEG, whereas its GSH content surged initially and then declined sharply along this PEG gradient (Figure 5a,b). At 10% and 15% PEG, AsA and GSH contents in *S. daiguii* were consistently higher than that in *S. meiliensis* (Figure 5a,b). These results suggested that *S. daiguii* had a greater advantage over *S. meiliensis* in accumulating these two non-enzymatic antioxidants under these two PEG-induced drought stress conditions.

### 2.7. Effects of PEG-Induced Drought Stress upon Antioxidant Enzyme Activity

**Figure 6:**
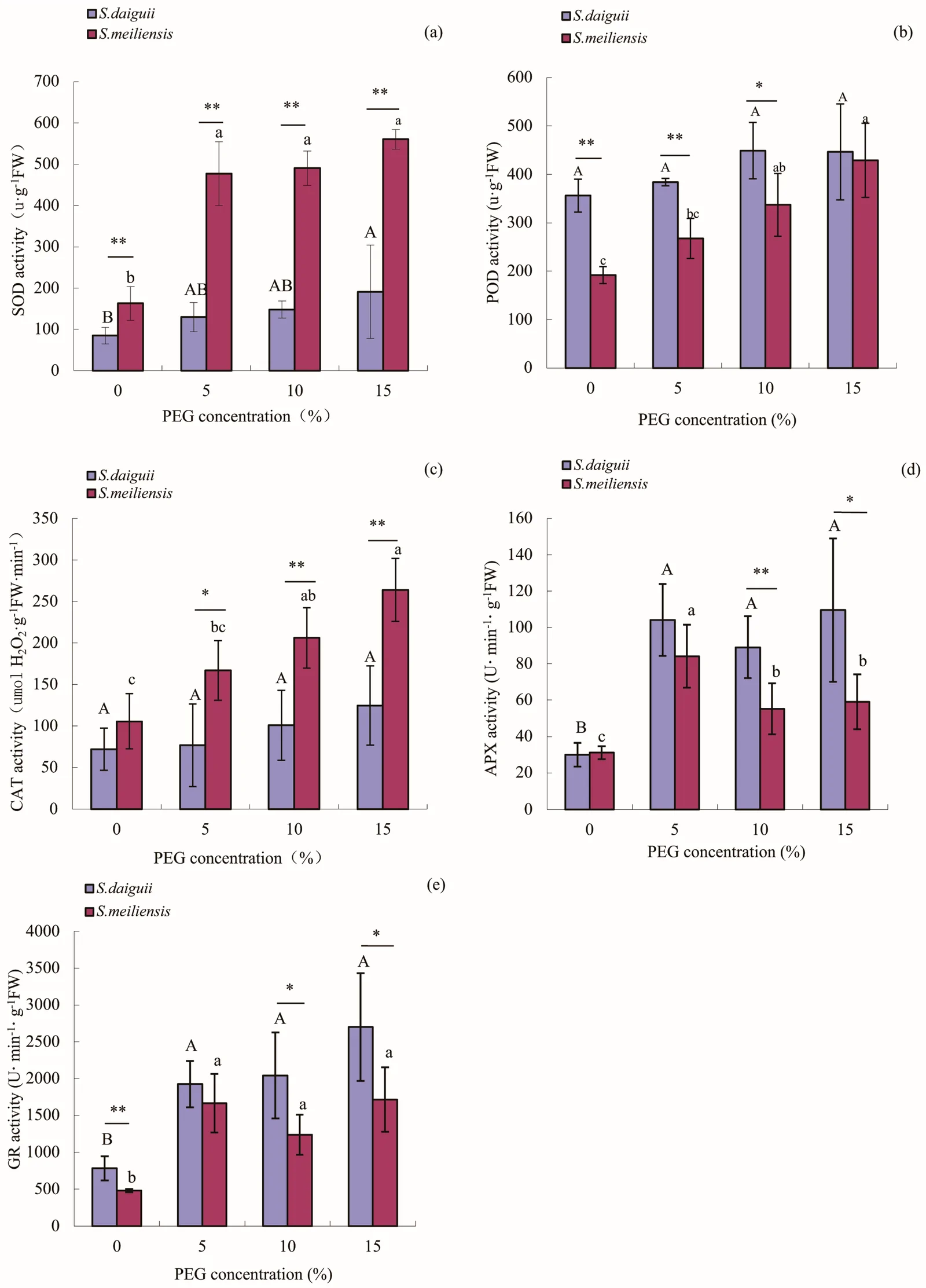
Response patterns of antioxidant enzyme activities to PEG-induced drought stress in *S. daiguii* and *S. meiliensis*. (**a**) SOD activity (SOD: superoxide dismutase); (**b**) POD activity (POD: peroxidase); (**c**) CAT activity (CAT: catalase); (**d**) APX activity (APX: ascorbate peroxidase); (**e**) GR activity (GR: glutathione reductase). Results are given as mean ± SE (*n* = 5). Distinct uppercase and lowercase letters show significant differences (*p* < 0.05) among various drought treatments for *S. daiguii* and *S. meiliensis*, respectively, as determined by the Tukey test. Single and double asterisks represent differences at * *p* < 0.05 and ** *p* < 0.01 between the two species under identical drought conditions, as analyzed by the independent-samples *t*-test. FW: fresh weight.

SOD activity in *S. meiliensis* reached a plateau at 5% PEG and then maintained this level at 10% and 15% PEG (Figure 6a). With increasing PEG concentrations, POD, and CAT activities in *S. meiliensis* exhibited a steady upward trend (Figure 6b,c). By contrast, its APX activity rose sharply to a peak at 5% PEG and slightly declined at 10% and 15% PEG, while still remaining markedly higher than the 0% PEG control (Figure 6d). These results suggested that *S. meiliensis* could activate these four antioxidant enzymes in response to the three tested PEG-induced drought stress conditions. In contrast, the four antioxidant enzymes in *S. daiguii* showed divergent response patterns under PEG-induced drought stress. Specifically, APX activity in *S. daiguii* reached a plateau at 5% PEG and then maintained this level at 10% and 15% PEG (Figure 6d). Only its SOD activity was significantly increased at 15% PEG (Figure 6a), while its POD and CAT activities showed no induced elevation across all tested PEG levels (Figure 6b,c).

Notably, SOD and CAT activities in *S. meiliensis* were consistently higher than those in *S. daiguii* at 5%, 10%, and 15% PEG (Figure 6a,c). These findings indicated that *S. meiliensis* had a greater advantage over *S. daiguii* in activating SOD and CAT under these three drought stress conditions. On the contrary, POD and APX activities in *S. daiguii* exceeded those in *S. meiliensis* at 10% PEG (Figure 6b,d), suggesting that *S. daiguii* was superior to *S. meiliensis* in POD and APX activation under this drought stress condition.

GR activity in *S. daiguii* reached a plateau at 5% PEG and then maintained this level at 10% and 15% PEG (Figure 6e). In contrast, GR activity in *S. meiliensis* showed an oscillating upward trend along this PEG gradient (Figure 6e). These results suggested that both *Salvia* species could accelerate GSH regeneration by enhancing GR activity in response to the three tested PEG-induced drought stress conditions. Notably, under 10% and 15% PEG treatments, GR activity in *S. daiguii* was consistently higher than that in *S. meiliensis* (Figure 6e), which indicated that *S. daiguii* had a greater advantage over *S. meiliensis* in promoting GSH regeneration under these two PEG-induced drought stress conditions.

### 2.8. Impacts of PEG-Induced Drought Stress upon Osmolyte Content

**Figure 7:**
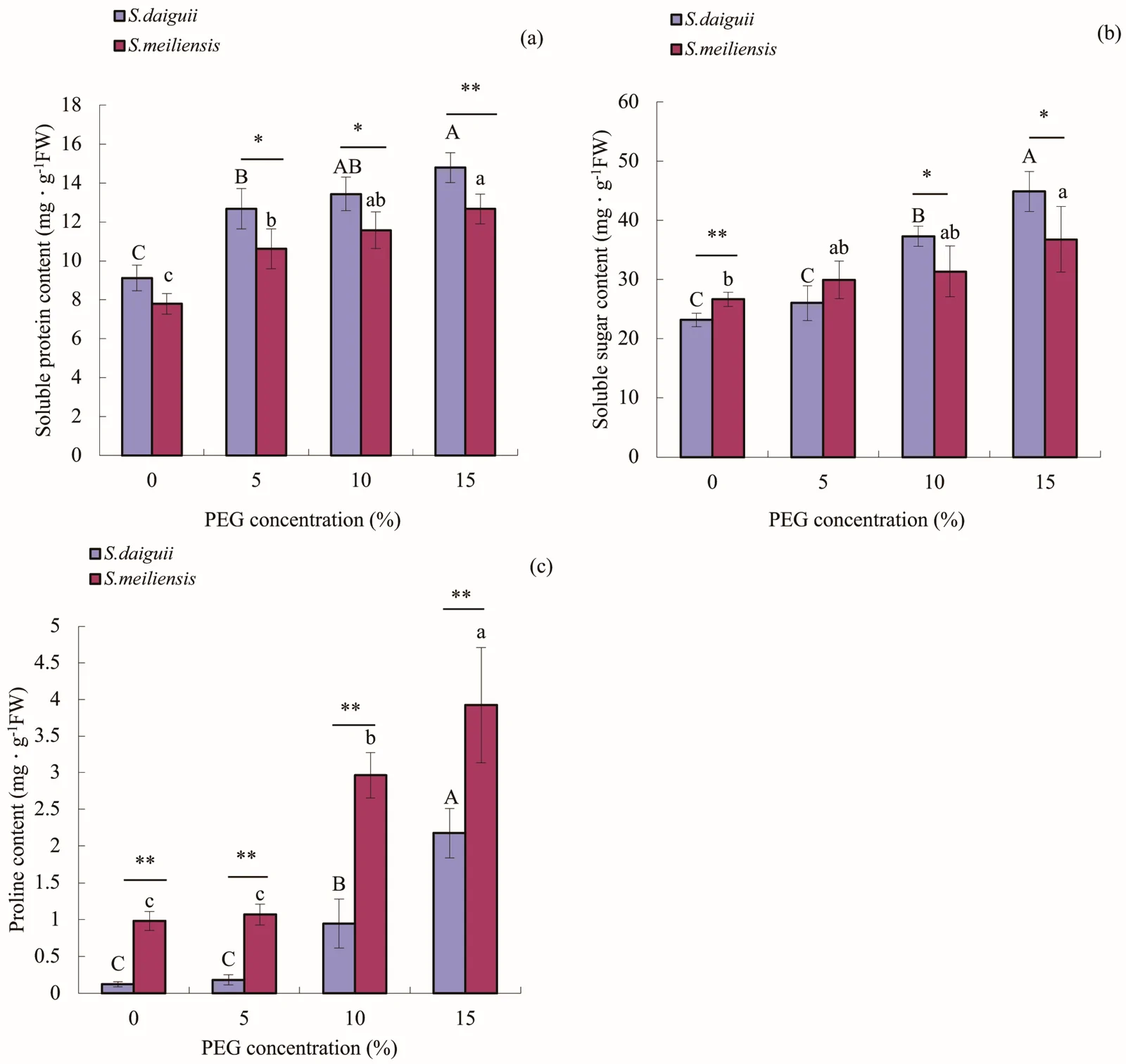
Response patterns of osmolyte content to PEG-induced drought stress in *S. daiguii* and *S. meiliensis*. (**a**) Soluble protein content; (**b**) Soluble sugar content; (**c**) Proline content. Results are given as mean ± SE (*n* = 5). Distinct uppercase and lowercase letters show significant differences (*p* < 0.05) among various drought treatments for *S. daiguii* and *S. meiliensis*, respectively, as determined by the Tukey test. Single and double asterisks represent differences at * *p* < 0.05 and ** *p* < 0.01 between the two species under identical drought conditions, as analyzed by the independent-samples *t*-test. FW: fresh weight.

With increasing PEG concentrations, the content of soluble protein, soluble sugar, and proline in *S. daiguii* and *S. meiliensis* exhibited upward trends (Figure 7a–c). These results suggested that both *Salvia* species could accumulate these osmolytes to cope with PEG-induced drought stress.

Compared to *S. meiliensis*, *S. daiguii* accumulated higher levels of soluble protein at 5%, 10%, and 15% PEG (Figure 7a). Likewise, *S. daiguii* accumulated more soluble sugar than *S. meiliensis* at 10% and 15% PEG (Figure 7b). These findings indicated that *S. daiguii* was superior in accumulating soluble protein and soluble sugar when subjected to moderate and severe drought stress (10% and 15% PEG). In contrast, *S. meiliensis* accumulated higher proline content than *S. daiguii* at 5%, 10%, and 15% PEG (Figure 7c), suggesting that *S. meiliensis* was superior in proline accumulation across all PEG-induced drought stress levels.

## 3. Discussion

### 3.1. Drought Tolerance Comparison Between S. daiguii and S. meiliensis

Drought stress typically reduces the relative water content in plants, causing loss of cell turgor and visible injury symptoms, including leaf curling, wilting, and abscission [51,52]. Thus, these phenotypic symptoms are often graded to generate drought injury indices, which are widely adopted as useful physiological indicators for evaluating plant drought tolerance [53,54,55]. Here, drought injury indices of *S. daiguii* were significantly lower than those of *S. meiliensis* across 5%, 10%, and 15% PEG (Table 1), indicating higher drought tolerance in *S. daiguii* under these tested PEG-induced drought conditions. In addition, drought stress also frequently triggers peroxidative damage in plant cells [56]. Accordingly, TBARS, an indicator of membrane lipid peroxidation, can serve as a reliable marker for evaluating plant drought tolerance [57]. In this study, TBARS content in *S. daiguii* was significantly lower than that in *S. meiliensis* across 5%, 10%, and 15% PEG (Figure 2), further suggesting higher drought tolerance in *S. daiguii* under these PEG-induced drought stress conditions.

### 3.2. Adaptive Mechanisms of S. daiguii in Response to Drought Stress

To clarify how *S. daiguii* achieves superior drought tolerance, we analyzed its adaptive mechanisms, which involve multi-faceted and coordinated regulation of osmotic balance, respiratory metabolism, antioxidant defense, and photosynthetic systems. For osmotic regulation, drought stress lessens the water potential difference between plant roots and soils, inhibiting passive water transport from soil to roots and ultimately causing water deficit [58]. To restore water absorption, drought-tolerant plants have evolved specialized osmotic regulation systems, typically by accumulating osmolytes including proline and soluble sugar to lower cellular osmotic potential [22,59]. Furthermore, drought-induced soluble proteins exhibit strong hydrophilicity and can act as auxiliary osmolytes to participate in osmotic adjustment of plants under water deficit [60,61]. For instance, soluble protein content increases markedly in rice seedlings upon drought treatment, which collaborates with soluble sugar to reduce leaf osmotic potential and preserve cell turgor pressure [60]. Moreover, *Castanopsis fissa* enhances the synthesis of soluble protein to strengthen its osmotic buffering capacity under drought stress [61]. Here, both *Salvia* species exhibited active osmotic adjustment under drought stress, yet they adopted divergent osmotic adaptive strategies. *S. daiguii* was superior in accumulating soluble sugar and soluble protein, while *S. meiliensis* showed an advantage in proline accumulation (Figure 7a–c).

Active water transport represents another important pathway through which plants absorb water from soil, and this process requires substantial energy from adenosine triphosphate (ATP) hydrolysis [38]. Notably, ATP generated by respiratory metabolism in leaves accumulates to higher levels and drives sucrose translocation from source leaves to roots, where sucrose functions as the principal substrate for root-local ATP synthesis fueling ATP-dependent active water uptake [62]. ATP generation relies on respiratory metabolic processes in mitochondria, which mainly include the TCA cycle and the ETC [38,63]. Commonly, drought is able to decelerate the TCA cycle by inhibiting respiratory enzymes in the mitochondrial matrix, thereby suppressing active water transport from soil to plant roots [38,64]. To counter this, drought-tolerant plants can activate respiratory enzymes regarding the TCA cycle under drought stress [26,36,37,38]. For example, drought-tolerant clones of coffee can activate MDH in leaves to accelerate the TCA cycle [65]. Moreover, exogenous NO enhances the activities of TCA cycle-associated enzymes FM and SDH in leaves, thereby promoting respiration in soybean plants under drought stress [66]. In this study, the activation of TCA cycle enzymes (FM, SDH, MDH) in the leaves was also observed in both *S. daiguii* and *S. meiliensis* at 10% and 15% PEG (Figure 4a–c). Notably, the activity of the three enzymes was markedly higher in *S. daiguii* than in *S. meiliensis* across 5–15% PEG, suggesting that *S. daiguii* has a greater advantage in restoring TCA cycle efficiency and ATP production under drought conditions, thus enhancing active water transport. As a terminal ETC enzyme, COX ensures efficient electron-oxygen binding for ATP generation [28], and elevated COX activity reduces electron leakage, alleviating ROS accumulation and peroxidative damage [67]. Here, COX activity in the leaves was significantly higher in *S. daiguii* than in *S. meiliensis* across all PEG treatments (Figure 4d), further confirming that *S. daiguii* has a greater advantage in restoring active water transport and alleviating peroxidative damage under drought conditions, which aligns with its lower TBARS content and milder aboveground water deficit (Figure 1a and Figure 2). Collectively, we consider that the superior performance of *S. daiguii* in activating TCA-cycle and terminal ETC enzymes is a key factor contributing to its greater relative drought tolerance when compared with *S. meiliensis*. It should be noted that only leaf respiratory enzyme activities were quantified rather than root respiratory intensity in the present study. Undoubtedly, root respiration acts as the primary energy source directly sustaining root active water transport [68], and quantitative measurement of root respiration rate will be a key direction for future studies to systematically clarify the energy supply mechanism underlying water absorption in *S. daiguii* and *S. meiliensis* under drought stress.

Beyond the respiratory chain enzymes, drought-tolerant plants can also activate a suite of antioxidant enzymes, such as CAT, POD, SOD, and APX, to scavenge excess ROS triggered by drought stress [22]. In this study, *S. meiliensis* and *S. daiguii* exhibited markedly different activation patterns and magnitudes for these four antioxidant enzymes. Specifically, *S. daiguii* had a clear advantage in activating POD and APX under the 10% PEG treatment, whereas *S. meiliensis* was superior in activating SOD and CAT under the same stress condition (Figure 6a–d). This difference indicates that the two species rely on different enzymatic antioxidant pathways to scavenge drought-induced ROS.

Many drought-tolerant plants can accelerate the biosynthesis of non-enzymatic antioxidants, including GSH and AsA, to scavenge drought-induced excess ROS [40,41,42]. In this study, only *S. daiguii* exhibited the capacity for sustained AsA and GSH biosynthesis along the increasing PEG-stress gradient (Figure 5a,b). Moreover, the content of these two non-enzymatic antioxidants was markedly higher in *S. daiguii* than in *S. meiliensis* under 10% and 15% PEG (Figure 5a,b), indicating a stronger non-enzymatic antioxidant capacity in *S. daiguii* under moderate-to-severe drought. ROS scavenging via GSH requires substantial consumption of this antioxidant. Interestingly, many drought-tolerant plants can activate GR to regenerate GSH under drought stress [40,42]. Here, both *S. daiguii* and *S. meiliensis* were capable of activating GR under drought stress (Figure 6e). Nevertheless, the magnitude of GR-activity up-regulation differed markedly under moderate and severe drought (10% and 15% PEG). GR activity increased to a greater extent in *S. daiguii*, enabling GSH regeneration to sufficiently offset GSH consumption and resulting in net GSH accumulation as well as high antioxidant capacity. By contrast, such an increase was limited in *S. meiliensis*. Consequently, GSH regeneration could not keep pace with consumption, leading to a substantial decline in GSH content and antioxidant capacity (Figure 5b and Figure 6e). A comparable phenomenon has been documented in tomato seedlings subjected to arsenite stress [59]. Therefore, we regard this as another key factor conferring stronger relative drought tolerance in *S. daiguii* when compared with *S. meiliensis*.

In photosynthetic systems, Chl *a* and Chl *b* are crucial photosynthetic pigments whose content serves as a credible index for evaluating plant photosynthetic performance. Drought-induced ROS can damage the photosynthetic apparatus, decreasing chlorophyll levels and photosynthate accumulation [22,69]. Nonetheless, many drought-tolerant plants can elevate chlorophyll levels to maintain stable photosynthesis under water-deficit conditions [70,71,72]. Here, a clear increasing trend in total chlorophyll content was also observed in both *S. daiguii* and *S. meiliensis* with increasing PEG concentrations (Figure 3c). Notably, *S. daiguii* maintained greater photosynthetic pigment stability and productivity than *S. meiliensis* under drought stress, as evidenced by its higher total chlorophyll content across all PEG-induced stress conditions (Figure 3c).

Chl *a* and Chl *b* perform distinct roles: Chl *a* captures light and initiates photochemistry, whereas Chl *b* serves solely as an accessory pigment to broaden the absorption spectrum [73,74]. The Chl *a*/*b* ratio therefore reflects the equilibrium between light absorption capacity and electron-excitation efficiency [75]. In drought-sensitive plants this ratio usually drops sharply, while tolerant species show slight decreases, stability or even increases [76], making the ratio a reliable tolerance index. Here, *S. daiguii* maintained a stable Chl *a*/*b* ratio across all PEG treatments (Figure 3d), which was conducive to coordinating light- harvesting and subsequent energy utilization within the photosynthetic apparatus. In contrast, the ratio declined progressively in *S. meiliensis* (Figure 3d), implying excess harvested light that may not be converted to chemical energy, potentially causing damage to the plant photosystems [75]. Collectively, the findings suggest that *S. daiguii* exhibits stronger homeostatic regulation of photosynthetic pigment composition under drought stress.

Taken together, *S. daiguii* and *S. meiliensis* adopt distinct osmotic-adjustment strategies in response to drought stress. The interspecific difference in drought tolerance between the two species is mainly determined by multiple physiological processes. Compared with *S. meiliensis*, *S. daiguii* exhibits higher photosynthetic pigment stability and productivity, which enables it to coordinate the balance between light harvesting and energy utilization under drought conditions. Furthermore, its superior performance in activating TCA-cycle and terminal ETC enzymes confers greater energy supply for water uptake under drought conditions. Meanwhile, its stronger non-enzymatic antioxidant capacity, especially the GR-dependent GSH regeneration capacity, enables more efficient scavenging of ROS induced by moderate and severe drought. Under these two drought conditions, higher APX and POD activities in *S. daiguii* further enhance its antioxidant capacity.

### 3.3. Implications of Drought Tolerance Assessment for S. daiguii Reintroduction

Multiple factors, including interspecific niche competition, abiotic stress, and human-caused disturbance, threaten wild populations of endangered plants and should be considered before conservation actions [5]. Previous work indicated that weak niche competitiveness and anthropogenic disturbance in native habitats represent major threats to *S. daiguii* [44], bringing uncertainties for in situ reintroduction within its original range. Accordingly, alternative ex situ sites are proposed as potential recipient locations. Under ongoing climate change, drought tolerance of propagules is a key pre-assessment index for successful reintroduction, and insufficient drought-tolerance evaluation has caused many reintroduction failures [5]. Our results suggest that *S. daiguii* possesses favorable drought tolerance capacity shaped by adjustments in photosynthetic, respiratory, and antioxidant systems. Accordingly, drought stress may not constitute a primary limiting factor when transferring ex situ propagated materials to alternative recipient sites. The present study provides preliminary physiological reference information supporting future reintroduction attempts for this endangered species.

Nevertheless, we acknowledge that PEG-4000 produces rapid osmotic stress under hydroponic conditions and cannot fully mimic the gradual soil-drying process, complex root development, and rhizosphere microbiome interactions under real-world field environments. Further field-based trials are required to validate the observations obtained from PEG-simulated drought treatments.

## 4. Materials and Methods

### 4.1. Plant Materials

Mature seeds of *S. daiguii* and *S. meiliensis* were collected from plants that had been ex situ conserved for three years at Shanghai Chenshan Botanical Garden. Seeds were sown in a 1:1 (m/m) peat:vermiculite mixture in pots (10 cm diameter, 15 cm height). After thorough watering, the pots were put in a phytotron in the dark at 25 °C with a relative humidity of 60%~70%, and ventilation was conducted once a day. After seed germination, the seedlings entered the cultivation stage, where each pot was quantitatively irrigated with 100 mL of water per week. The parameters of the phytotron were adjusted as follows: relative humidity of 40%~60%, photoperiod of 16/8 h light/dark, light intensity of 11,000 lux, and ventilation twice a day for 30 min each time. After 1 month of cultivation, seedlings (≈5 cm tall, four true leaves) of both species were selected for drought stress treatment.

### 4.2. Evaluating Plant Drought Tolerance

To assess plant drought tolerance, a simulated drought stress trial was conducted. Polyethylene glycol (PEG) 4000 was dissolved in Hoagland solution to prepare 5%, 10%, and 15% PEG solutions. Seedlings of *S. daiguii* and *S. meiliensis* were individually cultured in 500 mL of each PEG solution as the treatment groups. Hoagland solution without PEG (0%) served as the control, with seedlings of the two species cultured in 500 mL of this solution under the same conditions. Solutions for both treatments and the control were renewed every four days (on days 4, 8, and 12 of the treatment period). Ten biological replicas were set for each treatment and the control group. The growth performance of the two *Salvia* species was recorded daily, including the curling of leaf tips, leaf margins, and entire leaves. After 14 days of treatment, all seedlings were harvested. The drought injury index was scored on a 0–4 scale for individual plants, based on the percentage of damaged leaves and the severity of leaf wilting or curling. Specifically, the scoring grades were defined as follows: 0: All leaves of the plant were normal and turgid, with no visible wilting, curling, or drought injury symptoms. 1: ≤25% of leaves showed slight wilting or upward curling at leaf tips, with normal plant vigor. 2: 26–50% of leaves exhibited obvious wilting and upward curling at both tips and margins, with slightly reduced plant growth. 3: 51–75% of leaves displayed marked upward curling across the entire leaf blade, with partial yellowing or drying at leaf tips/margins. 4: More than 75% of leaves were seriously wilted and curled upward throughout the blade, accompanied by obvious desiccation and partial leaf abscission or impending abscission [53,54,55]. The relative water content of aboveground and belowground tissues was then determined using the drying-weighing method [77].

To investigate the physiological systems underlying drought tolerance, a second simulated drought stress assay was performed as described above. All seedlings were harvested and frozen at −80 °C prior to physiological analysis of drought tolerance.

### 4.3. Parameter Determination

#### 4.3.1. Determination of TBARS

The thiobarbituric acid (TBA) method was used to determine the content of TBARS [78]. In brief, 0.25 g leaf tissue was pulverized using liquid nitrogen (LN) and homogenized in 2.5 mL pre-cooled phosphate buffer (50 mM, pH 7.8), the homogenate was centrifuged at 12,000× *g* for 20 min, and the resulting supernatant was collected and used as the TBARS assay solution. A reaction mixture containing 2 mL assay solution and 2 mL TBA solution was incubated in a water bath (100 °C) for 40 min. At room temperature (RT), chloroform was added, and the mixture was vortexed vigorously and left to stand for 15 min for phase separation. The absorbance of the upper aqueous phase was measured at 538 nm. The content of TBARS was expressed as μmol g^−1^ FW.

#### 4.3.2. Determination of Chlorophyll

Chlorophyll extraction was performed following the acetone-ethanol mixture approach of Wellburn. [79]. In short, 0.2 g leaf tissue was homogenized in a pre-cooled mortar with quartz sand and 2 mL extraction solution (acetone: anhydrous ethanol = 1:1, *v*/*v*). The resulting homogenate was filtered through quantitative filter paper. The residue and mortar were rinsed with the extraction solution for complete recovery. The combined filtrate and rinsings were adjusted to a final volume of 10 mL. The extract absorbance was measured at 645 and 663 nm. The levels of Chl *a*, Chl *b*, and total chlorophyll were calculated via standard formulas [80].

#### 4.3.3. Analyses of Key Enzymes in TCA Cycle and Respiratory Chain

In the TCA cycle and respiratory chain, the activities of four important enzymes, including FM, SDH, MDH, and COX, were determined using a spectrophotometric method.

For analysis of FM activity, 0.25 g leaf samples were combined with 2.5 mL extraction buffer (1 mM ethylenediaminetetraacetic acid (EDTA); 1 mM MgCl_2_; 5 mM dithiothreitol (DTT); 10 mM KCl; 150 mM Tricine-KOH, pH 7.5). The mixture was homogenized on ice and then centrifuged at 15,000× *g* for 5 min at 4 °C. The resulting supernatant was collected for subsequent enzyme activity assay [81]. The enzyme activity was spectrophotometrically determined by mixing 25 μL enzyme extract with 2 mL assay medium (5 mM magnesium chloride; 50 mM malic acid; 50 mM potassium phosphate buffer, pH 7.0) and recording the absorbance change at 240 nm [82]. One unit (U) of FM activity was defined as the amount of enzyme required to oxidize 1 μmol fumaric acid per minute, and the activity was expressed as U g^−1^ FW.

To determine the SDH activity, 0.2 g leaf tissue was homogenized on ice in 2 mL extraction buffer (1 mM EDTA; 1 mM MgCl_2_; 10 mM KCl; 50 mM Tris-HCl, pH 7.5). At 4 °C, the homogenate was centrifuged at 10,000× *g* for 10 min, and the resulting supernatant was collected as the enzyme solution [83]. The enzyme activity was determined spectrophotometrically by mixing 0.1 mL enzyme solution with 3 mL assay medium (8 μM 2,6-dichlorophenolindophenol (DCPIP); 0.1 mM phenazine methosulfate; 1 mM sodium azide (NaN_3_); 10 mM succinate; 50 mM potassium phosphate buffer, pH 7.8), and recording the immediate reduction in absorbance at 600 nm [84]. One u of SDH activity was defined as the amount of enzyme required to cause a reduction in absorbance of 0.01 at 600 nm per minute, and the activity was expressed as u g^−1^ FW.

For the analysis of MDH activity, a 0.2 g sample was ground to a fine powder under LN and homogenized with 2 mL extraction buffer (0.2 mM MgCl_2_; 2 mM DTT; 10% (*v*/*v*) glycerol, 100 mM Tris-HCl, pH 7.5). At 4 °C, the mixture was incubated for 1 h and subsequently centrifuged at 12,000× *g* for 10 min; the supernatant was collected as the crude enzyme solution [85]. The activity was determined spectrophotometrically by mixing 0.1 mL crude enzyme solution with 2 mL assay medium (50 mM Tris-HCl, pH 7.5; 0.15 mM reduced nicotinamide adenine dinucleotide (NADH); 4 mM oxaloacetic acid), and recording the reduction in absorbance at 340 nm (reflecting NADH consumption) during 3 min [86]. One U of MDH activity was defined as the amount of enzyme required to oxidize 1 μmol NADH per minute, and the activity was expressed as U g^−1^ FW.

For the analysis of COX activity, 1 g leaf tissue was ground to a fine powder under LN and homogenized in 1 mL ice-cold extraction buffer (0.1 M Tris-citrate buffer, pH 8.0; 5 mM EDTA; 1 μL·mL^−1^ β-mercaptoethanol). The homogenate was incubated on ice for 2 min; after filtration, it was centrifuged at 12,000× *g* for 10 min at 4 °C. The supernatant was collected as the crude enzyme. The enzyme activity was determined spectrophotometrically by mixing 20 μL crude enzyme with 2 mL reaction medium (20 μM reduced cytochrome c; 0.2 M sucrose; 50 mM phosphate buffer, pH 7.1), and monitoring the reduction in absorbance at 550 nm [87]. One U of COX activity was defined as the amount of enzyme that oxidized 1 μmol reduced cytochrome c per minute, and the specific activity was expressed as U g^−1^ FW.

#### 4.3.4. Determination of Non-Enzymatic Antioxidants

AsA and GSH were obtained from 0.4 g sample of fresh leaves, which was homogenized in 4 mL pre-cooled 5% (*w*/*v*) sulfosalicylic acid. The homogenate was centrifuged at 16,000× *g* for 20 min at 4 °C. The supernatant was collected for subsequent analysis [88].

For the determination of AsA content, 300 μL supernatant was mixed with 300 μL of 50 mM sodium dihydrogen phosphate buffer (pH 7.4) and 300 μL distilled water, followed by incubation at RT for 15 min. Subsequently, 150 μL of 0.5% (*w*/*v*) N-ethylmaleimide, 600 μL of 10% (*w*/*v*) trichloroacetic acid, 600 μL of 44% (*v*/*v*) phosphoric acid (H_3_PO_4_), 600 μL of 14% (*w*/*v*) bipyridyl (dissolved in 70% (*v*/*v*) ethanol), and 300 μL of 3% (*w*/*v*) FeCl_3_ were added sequentially. After vortexing, the mixture was incubated for 1 h at 37 °C. The absorbance at 525 nm was measured.

The content of GSH was determined according to Griffith and Tate with modifications [89,90]. Briefly, 200 μL of the supernatant was mixed with 400 μL phosphate buffer (125 mM, pH 7.8), 200 μL 5,5′-dithio-bis-2-nitrobenzoic acid solution (DTNB, 6 mM), 1 mL reduced nicotinamide adenine dinucleotide phosphate solution (NADPH, 0.3 mM), and 200 μL sterile distilled water. The absorbance of the reaction mixture was recorded at 412 nm.

#### 4.3.5. Analyses of Antioxidant Enzyme Activity

SOD, POD, and CAT were extracted from 0.4 g of plant tissues, which were ground into a fine powder under LN and homogenized in 2 mL extraction buffer (100 mM sodium phosphate buffer, pH 7.0, comprising 1 mM AsA and 0.5% (*w*/*v*) PVP). Following filtration, the homogenate was centrifuged at 5000× *g* (4 °C, 15 min), and the supernatant was collected as the crude enzyme extract for subsequent assays [91].

Nitroblue tetrazolium (NBT) photochemical reduction was used for SOD activity analysis. Initially, 100 μL crude enzyme solution was transferred into 3 mL premixed reaction solution. The solution was formulated with 100 mM sodium phosphate buffer (pH 7.6), 0.1 mM EDTA, 12 mM L-methionine, 50 mM sodium carbonate, 10 μM riboflavin, and 50 μM NBT. The mixture was illuminated under 4000 lx light for 20 min to initiate the reaction, which was immediately terminated by placing the samples in darkness. The absorbance was measured at 560 nm spectrophotometrically [91]. One u of SOD activity was defined as the amount of enzyme required to inhibit the photochemical reduction of NBT by 50%, and the activity was expressed as u g^−1^ FW.

For the analysis of POD activity, the guaiacol method was used. A 100 μL aliquot of the crude enzyme extract was mixed with 3 mL reaction mixture (50 mM potassium phosphate buffer, pH 6.0, containing 20 mM guaiacol and 0.01% (*v*/*v*) hydrogen peroxide (H_2_O_2_)). The absorbance at 470 nm was monitored spectrophotometrically at 0, 1, 2, and 3 min [92]. One u of POD activity was defined as the amount of enzyme required to cause an increase in absorbance of 0.01 per minute at 470 nm, and the activity was expressed as u g^−1^ FW.

CAT activity was assayed following the titrimetric method modified from Kar and Mishra [93]. A 5 mL reaction mixture contained 200 μL crude enzyme solution, 3 mL phosphate buffer (300 μM, pH 6.8), 1 mL 100 μM H_2_O_2_ substrate, and 0.8 mL distilled water. After incubating the mixture at room temperature for 1 min, 10 mL sulfuric acid (2%, *v*/*v*) was added to terminate the enzymatic reaction. The residual H_2_O_2_ was titrated with 2 mM KMnO_4_ solution until a faint purple hue developed and persisted for at least 30 s. Parallel blank controls were prepared simultaneously without incubation, and sulfuric acid was added immediately to terminate the reaction at time zero. The CAT activity was expressed as μmol H_2_O_2_ decomposed per gram fresh weight per minute (μmol g^−1^ FW min^−1^).

For the analysis of APX activity, 0.3 g sample was ground to a fine powder in LN and homogenized in 0.75 mL extraction buffer (50 mM potassium phosphate buffer, pH 7.0, comprising 1 mM AsA and 1 mM EDTA). The homogenate was filtered through four layers of gauze and centrifuged at 10,000× *g* for 10 min at 4 °C, and the resulting supernatant was collected as the crude enzyme solution. The enzyme activity was determined by mixing 2.5 mL assay medium (0.5 mM AsA; 50 mM potassium phosphate buffer, pH 7.0) with 0.1 mL crude enzyme solution. The reaction was activated by adding hydrogen peroxide (H_2_O_2_) to a final concentration of 0.1 mM, and the immediate reduction in absorbance at 290 nm was recorded [94]. One U of APX activity was defined as the amount of enzyme required to oxidize 1 μmol AsA per minute, and the activity was expressed as U g^−1^ FW.

The activity of GR, a key antioxidant cycle enzyme, was determined using a spectrophotometric method. Briefly, 0.2 g sample was ground to a fine powder in LN. The obtained powder was mixed thoroughly in 0.6 mL extraction buffer, which contained 1 mM EDTA, 1% (*w*/*v*) polyvinylpyrrolidone (PVP), and 100 mM potassium phosphate buffer adjusted to pH 7.5. The resulting homogenate underwent centrifugation at 10,000× *g* for 30 min at 4 °C, and 10 mM DTT was added into the resulting supernatant, before thorough mixing for the subsequent enzyme activity assay. The enzyme activity was determined by mixing 0.1 mL of the crude enzyme solution with 2.5 mL assay medium (2 mM EDTA; 100 mM potassium phosphate buffer, pH 7.8). The reaction was stimulated by adding NADPH and GSSG to a final concentration of 0.2 mM and 0.5 mM, respectively. The decrease in absorbance at 340 nm was recorded for 5 min [95]. One U of GR activity was defined as the amount of enzyme required to oxidize 1 μmol NADPH per minute, and the activity was expressed as U g^−1^ FW.

#### 4.3.6. Determining Osmotic Adjustment Substances

Soluble sugar content was tested by the anthrone colorimetric method [96]. Briefly, 0.2 g leaf sample was homogenized in 5 mL deionized water, and the homogenate was heated in a boiling water bath for 30 min. At RT, the mixture centrifuged at 8000× *g* for 10 min. The obtained supernatant was collected, adjusted to a final volume of 50 mL with deionized water, and used as the soluble sugar assay solution. For the color reaction, 1.0 mL assay solution and 4.0 mL anthrone reagent were mixed to form the reaction mixture, which was heated in a water bath (100 °C) for 10 min. Upon cooling, the absorbance was measured at 620 nm. Soluble sugar content was quantified against a standard curve and expressed as mg g^−1^ FW.

Soluble protein content was determined using the Coomassie Brilliant Blue G-250 staining method [97]. Briefly, 0.2 g leaf tissue was homogenized in 5 mL deionized water, and the homogenate was thoroughly vortexed before centrifugation at 8000× *g* for 10 min at 4 °C. The obtained supernatant was used as the soluble protein assay solution. A reaction mixture containing 1.0 mL assay solution and 4.0 mL Coomassie Brilliant Blue G-250 reagent was vortexed and incubated at RT for 2–5 min, followed by absorbance measurement at 595 nm. The content of soluble protein was determined using a standard curve, with results expressed as mg g^−1^ FW.

Proline content was tested by the acid ninhydrin method [98]. Specifically, 5 mL of 3% (*w*/*v*) sulfosalicylic acid was added to 0.2 g of leaf tissue for homogenization. Following a 10 min heating treatment at 100 °C, the homogenate was centrifuged at 8000× *g* for 5 min. The supernatant was gathered as the proline assay solution. The color reaction was initiated by combining proline assay solution, glacial acetic acid, and acidic ninhydrin reagent in volumes of 2 mL, 2 mL, and 3 mL, respectively. The resultant mixture was kept at 100 °C for 40 min. After cooling, toluene (4 mL) was added, followed by vigorous vortexing to collect the red chromophore. Absorbance of the upper organic phase was read at 520 nm. Proline concentration was determined from the standard curve and reported as mg g^−1^ FW.

### 4.4. Statistical Analysis

Statistical analysis was performed using Microsoft Excel 2016 and IBM SPSS Statistics 27.0. Prior to analysis, Levene’s test was conducted to assess the homogeneity of variances. One-way analysis of variance (ANOVA) was used to evaluate differences among various treatments within the same species, followed by the Tukey test at the *p* < 0.05 significance level. An independent-samples *t*-test was employed to compare significant differences between the two species under the same treatment, with the same significance level. All data are shown as mean ± standard error (SE) of five independent biological replicates.

## 5. Conclusions

This study clarified the drought tolerance and underlying physiological mechanisms of the endangered *Salvia daiguii*, using *S. meiliensis* as a reference. Under PEG-induced drought stress, *S. daiguii* exhibited stronger drought tolerance, characterized by lower drought injury indices, less TBARS accumulation, and higher relative water content. This tolerance advantage derives from integrated modulation of photosynthetic, osmotic-regulatory, antioxidant, and respiratory systems, including maintenance of homeostatic photosynthetic pigment composition, optimized osmolyte accumulation, enhanced antioxidant capacity, and activated respiratory enzymes. These results, obtained under PEG-induced drought stress conditions, suggest that drought stress is unlikely to limit the reintroduction of ex situ *S. daiguii* propagules, providing preliminary physiological insights to inform future field-based conservation and reintroduction trials.

## Figures and Tables

**Table 1:** Drought injury indices of two *Salvia* species under different PEG-induced drought stress levels.

PEG Concentration (%)	Drought Injury Indices		*p*-Value
	*S. daiguii*	*S. meiliensis*	
0	0.00 ± 0.00 B	0.10 ± 0.10 c	*p* = 0.331
5	0.10 ± 0.10 B	1.10 ± 0.18 b	*p* < 0.01 **
10	0.50 ± 0.17 AB	1.60 ± 0.16 b	*p* < 0.01 **
15	0.90 ± 0.18 A	2.40 ± 0.22 a	*p* < 0.01 **

Data are shown as the mean ± SE (*n* = 10). In the identical column, divergent uppercase and lowercase letters stand for significant differences (*p* < 0.05) in different PEG concentrations for *S. daiguii* and *S. meiliensis*, respectively, as measured by the Tukey test. Asterisks indicate highly significant differences (*p* < 0.01) between *S. daiguii* and *S. meiliensis* at the identical drought stress level, as measured by the independent-samples *t*-test.

## Data Availability

Data will be made available on request. The data are not publicly available due to inclusion in ongoing follow-up research.
